# Pectasol‐C Modified Citrus Pectin targets Galectin‐3‐induced STAT3 activation and synergize paclitaxel cytotoxic effect on ovarian cancer spheroids

**DOI:** 10.1002/cam4.2334

**Published:** 2019-06-13

**Authors:** Ghamartaj Hossein, Sina Halvaei, Yassaman Heidarian, Zeinab Dehghani‐Ghobadi, Mina Hassani, Homa Hosseini, Nima Naderi, Shahrzad Sheikh Hassani

**Affiliations:** ^1^ Department of Animal Biology, Developmental Biology Laboratory, College of Science University of Tehran Tehran Iran; ^2^ Department of Cell and Molecular Biology, Kish International Campus University of Tehran Kish Iran; ^3^ Neuroscience Research Center Shahid Beheshti University (Medical Sciences) Tehran Iran; ^4^ Department of Gynecology Oncology Valiasr Imam Khomeini Hospital, Tehran University of Medical Science Tehran Iran

**Keywords:** Galectin‐3, integrin, invasion, migration, ovarian cancer, STAT3

## Abstract

Here we sought to determine the relationship between STAT3 activity and Galectin‐3 (Gal‐3) and to investigate the cytotoxic effect of PectaSol‐C Modified Citrus Pectin (Pect‐MCP) as a specific competitive inhibitor of Galectin‐3 (Gal‐3) in combination with Paclitaxel (PTX) to kill the ovarian cancer cell SKOV‐3 multicellular tumor spheroid (MCTS). To this order, SKOV‐3 cells in 2D and 3D cultures were treated with exogenous Gal‐3 for the assessment of STAT3 activity. Two‐way ANOVA main effect and IC_50_ of each drug Paclitaxel (PTX) and Pect‐MCP or in combination were obtained from MTT assay results. The phosphorylated STAT3 levels, migration, invasion, integrin mRNA and p‐AKTser^473^ levels were assessed in the absence or presence of each drug alone or in combination. Gal‐3 expression levels were assessed in human serous ovarian cancer (SOC) specimens and its correlation with different integrin mRNA levels was further assessed. Our results showed that Gal‐3 expression level was significantly increased in MCTS compared to monolayer SKOV‐3 cells which triggered STAT3 phosphorylation. Moreover, Pect‐MCP synergized with PTX to kill SKOV3 MCTS through abrogation of STAT3 activity and reduced expression of its downstream target HIF‐1α, reduced integrin mRNA levels, and subsequently decreased AKT activity. There were higher expression levels of Gal‐3 in human high‐grade SOC specimens compared to the normal ovary and borderline SOC which positively and significantly correlated with α5, β2 and β6 integrin mRNA levels. Together, these results revealed for the first time that Pect‐MCP could be considered as a potential drug to enhance the PTX effect on ovarian cancer cells MCTS through inhibition of STAT3 activity.

## 1 INTRODUCTION

Ovarian cancer (OC) cells are disseminated throughout the abdominal cavity by peritoneal fluid or ascites and often form multilayer spheroid‐like structures which could attach to mesothelium, invade the peritoneum and initiate the metastatic tumor growth.^1^, ^2^ These spheroids in ascites are capable of tumorigenesis in vivo and are chemoresistant in vitro.^3^, ^4^

Galectin‐3 (Gal‐3) is a unique member of galectin family containing a C‐terminal carbohydrate recognition domain, which binds to β‐galactosides and its N‐terminal domain is needed for Gal‐3 enigmatic behavior and cross‐linking activity.^5^ Changes in Gal‐3 expression are commonly seen in cancer and pre‐cancerous conditions.^6^ Moreover, Gal‐3 has anti‐ or proapoptotic action depending on its subcellular localization and is involved in cellular proliferation, adhesion, motility, metastasis and thereupon tumor progression.^6^

Previous studies associated Gal‐3 expression with OC chemoresistance.^7^, ^8^ PectaSol‐C Modified Citrus Pectin (Pect‐MCP) is a derived from a water‐insoluble citrus pectin which becomes a soluble dietary supplement upon pH/temperature‐modification.^9^ Pect‐MCP act as a ligand for Gal‐3 due to its high content in β‐galactoside residues and impedes Gal‐3 interaction with its natural ligands.^9^

In normal cells, Signal transducer and activator of transcription 3 (STAT3) are transiently activated in response to specific growth factors and cytokines, while STAT3 is constitutively activated in many cancerous cells, including OC cells.^10^, ^11^ STAT3 activation is responsible for several key factors in tumor progression, involving uncontrolled cell proliferation, angiogenesis promotion and importantly facilitating chemoresistance.^12^ In most of the high‐grade serous OC, activated STAT3 (p‐STAT3 tyr^705^) was localized in the nucleus and was associated with increased chemoresistance and subsequent decreased patient survival.^13^ In addition, the sustained activation of the STAT3 pathway was demonstrated in cisplatin‐or paclitaxel‐treated OC cell lines.^14^

Although OC initially responds well to the first‐line chemotherapeutic agent Paclitaxel (PTX), it often relapses, and cancer cells become resistant to PTX.^15^ Concurrent use of two or more anticancer drugs provides an efficient approach by affecting distinct molecular targets and minimize drug resistance, toxicity, and side effects but only if the components of the drug combination have a different mechanism of action.^16^, ^17^

Multicellular tumor spheroid culture (MCTS) has been reported to be a more suitable candidate for studying drug penetration due to the high resemblance to solid tumors.^18^, ^19^ Here we sought to investigate the role of Gal‐3 in migration, invasion, and chemoresistance of SKOV3 MCTS and to understand its molecular mechanism. We further explored a possible synergistic effect of Pect‐MCP as a specific Gal‐3 competitive inhibitor in combination with PTX to kill ovarian cancer cell MCTS.

## 2 MATERIAL AND METHODS

### 2.1 Cell line and reagents

Human ovarian cancer SKOV‐3 cells were a kind gift from Dr AH Zarnani (Avicenna research center, Tehran, Iran). Cells were grown at 37°C in 5% CO_2_ atmospheres under 90%‐95% humidity in Roswell Park Memorial Institute (RPMI)‐1640 medium supplemented with 10% fetal bovine serum (FBS) and Penicillin/Streptomycin antibiotics obtained from Life Technologies GmbH (Darmstadt, Germany). Reagents were obtained from the following companies: Paclitaxel (Stratagene, Switzerland), rabbit polyclonal anti‐human phospho‐STAT3 Tyr705 (OriGene Technologies, Rockville, MD, USA); rabbit polyclonal anti‐STAT3 and anti‐GAPDH (Abcam, Cambridge, UK); rabbit polyclonal anti‐HIF1‐α (Santa Cruz Biotechnology, Inc., Texas, USA) and rabbit polyclonal antihuman Gal‐3 (ReliaTech, GmbH, Germany). Cleaved caspase‐3 colorimetric assay kit was purchased from GenScript (NJ, USA); Pectasol‐C Modified Citrus Pectin (Pect‐MCP) was obtained from EcoNugenics (Santa Rosa, CA, USA). Recombinant human galectin‐3 (rhGal‐3) was obtained from Pepro Tech (NJ, USA) and Matrigel was from BD Bioscience (San Jose, CA, USA). MTT and Methylcellulose were purchased from Sigma‐Aldrich (Saint‐Louis, USA).

### 2.2 Substrate‐dependent cell adhesion assay

Cells were treated with rhGal‐3 (3 µM) or Pect‐MCP (0.025%) alone for 24 hours then trypsinized and seeded on Matrigel‐coated 96 wells plates; adhesion assay was performed as previously described.^20^

### 2.3 MCTS culture and MTT assay

Uniformity in spheroid size and number of cells incorporated into a spheroid for effective drug penetration is important. To this order, we used here MCTS of less than 200 µm in diameter without necrotic core center. Drops of 30 µL containing 1000 SKOV3 cells suspended in RPMI with 10% FBS and 20% methylcellulose were placed on the inner side of the lid of 60 mm tissue culture dishes. After 3 days, SKOV‐3 MCTS (12 MCTS/well) was transferred into agarose coated 96‐well plates containing 10% FBS, then treated with PTX (0.1, 1, 5, 15, 20, 100 µM) or Pect‐MCP (0.025%) alone or in combination for 24 and 48 hours. To assess the effect of Gal‐3 on cell viability, MCTS were treated with rhGal‐3 (10, 100, 250, 500 ng/mL) in RPMI/FBS 0.5% for 48 hours and the main effect and IC_50_ of PTX or Pect‐MCP alone or in combination were assessed by MTT assay as previously described.^20^

### 2.4 Cleaved caspase‐3 activity and cell cycle analysis

The effect of rhGal‐3 on the viability and apoptosis of SKOV‐3 MCTS were assessed using MTT assay; colorimetric assay of caspase‐3 activity and cell cycle analysis were performed as previously described.^20^

### 2.5 Transwell migration and invasion assay and gelatin zymography

The effect of exogenous Gal‐3 or Pect‐MCP on cell migration and invasion was assessed in 2D as well as 3D SKOV‐3 cell cultures. To this order, monolayer SKOV‐3 cells were treated with rhGal‐3 (300 nM or 3 µM) or Pect‐MCP (0.025%), alone or in combination. After 24 hours, cells were trypsinized and migration and invasion assay was performed with transwells as previously described.^21^ SKOV3 MCTS were treated with PTX (5 µM) or Pect‐MCP (0.025%) alone or in combination for 24 hours. Then, MCTS were disaggregated by accutase, counted using trypan blue exclusion test and 25 × 10^3^ viable cells were seeded on the top of transwell chambers for cell migration/ invasion assay as previously described.^21^ The conditioned medium harvested from invasion assay was used for detection of MMP‐2 and ‐9 by gelatin zymography assay as previously described.^22^

### 2.6 Immunofluorescence and western blot

Cells were plated onto 6‐well plates, with or without rhGal‐3 (3 µM or 30 µM) for 30 minutes, and then fixed with ice‐cold 4% paraformaldehyde. The cells were permeabilized with PBS containing 0.1% Triton X‐100, blocked with 1% BSA (Sigma, Saint‐Louis, USA) and incubated overnight at 4°C with polyclonal rabbit anti‐human p‐STAT3 Tyr^705^ then washed and incubated with anti‐rabbit IgG‐FITC for 1h. DAPI (DAPI hydrochloride) was used to stain nuclei. Images were obtained with a Zeiss invert fluorescent microscope. The expression levels of total STAT3 (1:2000), p‐STAT3 Tyr^705^ (1:2000), HIF‐1α (1:1500), and GAPDH (1:2000) as internal control were assessed by western blot analysis.

### 2.7 Human ovarian surface epithelial cells isolation and immunohistochemistry

The effect of Pect‐MCP alone or in combination with PTX on primary normal ovarian surface epithelial cells (OSE) was further determined. To this order, ovarian tissues were obtained from healthy women undergoing oophorectomy (n = 2, 20‐25 years old) at Imam Khomeini University Hospital Complex with informed consent from patients. Ovarian tissues were treated with 1.7 U/mL of Dispase II (Gibco) in 35 mm Petri dish for 30 minutes at 37°C and the surface of ovary was gently scraped with fine rubber policeman, then the specimen was washed twice with PBS by transferring to other Petris dishe. Then MCDB/M199 medium +10% FBS was added to the pool of isolated normal OSE and centrifuged at 200 g for 10 minutes at room temperature. The cell pellet was suspended in MCDB/M199 medium +10% FBS and left for 3 weeks in culture. The morphology of isolated OSE was epithelial (Figure S1A) and they expressed epithelial markers (CDH1, EpCAM, KRT18, and KRT7) and vimentin^23^ (Figure S1B). Cells were trypsinized at 70%‐80% confluency and cells at passage 2 were used for 3D culture using hanging drop (1000 cells/drop). OSE spheroids (n = 10/well) were transferred into 96 wells plate and treated for 24 hours with PTX (0.01 to 100 µM) or Pect‐MCP (0.025%) alone or in combination and MTT assay was performed.

Gal‐3 and integrin expression level in serous ovarian cancer (SOC) specimens was assessed by immunohistochemistry (IHC) and qRT‐PCR, respectively. To this order, SOC subtypes and normal ovarian tissue specimens were obtained from surgeries performed at Imam Khomeini University Hospital Complex and informed consent was obtained from patients. All samples were examined by a pathologist for histological diagnosis and grade. The patients (age = 24‐70 and Median = 46,) were divided in four groups, namely: normal ovary (n = 10); borderline SOC (BLSOC, n = 12); low‐grade SOC (LGSOC, n = 12), and high‐grade SOC (HGSOC, n = 22). The characteristics of the patients were described in Table S1. Samples were chopped into small pieces of 50 mg with a surgical bladder and were immediately snap frozen in liquid nitrogen for further qRT‐PCR analysis or were processed for paraffin embedding and IHC analysis. Paraffin‐embedded ovarian tumor specimens were processed for IHC analysis as previously described.^21^ The antibody used for IHC was anti‐human Gal‐3 (1:200), the samples were analyzed semi‐quantitatively as negative (0%‐10%), 1+ or moderate (11%‐50% positive tumor cells) and 2+ or strong (>50% positive tumor cells).

### 2.8 RNA extraction and quantitative real‐time polymerase chain reaction

Total RNA from SKOV‐3 cells, as well as patient samples, was extracted using TRIzol reagent (Invitrogen, Paisley, UK) and qRT‐PCR was performed as previously described.^22^ The sequence of the primers used in this study is provided in Table S2. The expression levels of assessed genes were normalized relative to ACTB gene. Quantification of gene expression was performed via the standard curve method using REST‐RG software version 3.

### 2.9 Statistical analysis

Normality of nominal variables was analyzed by performing the Kolmogorov‐Smirnov test. Skewed and normal distributed metric variables were analyzed by Mann‐Whitney U and one‐way ANOVA tests, respectively, using SPSS version 16 (SPSS Inc, IL, USA). IC_50_ was determined by PHARM software and combined growth‐inhibitory effect for determining the main effect was performed by two‐way ANOVA and Bonferroni multiple comparisons tests that were run on Graph pad prism 5 (San Diego California, USA). All experiments were performed for at least three times; results were expressed as mean + SD and *P* ≤ 0.05 was considered statistically significant.

## 3 RESULTS

### 3.1 SKOV‐3 MCTS express higher Gal‐3 expression level compared to monolayer which is associated with increased MCTS viability, survival, and growth

**Figure 1:**
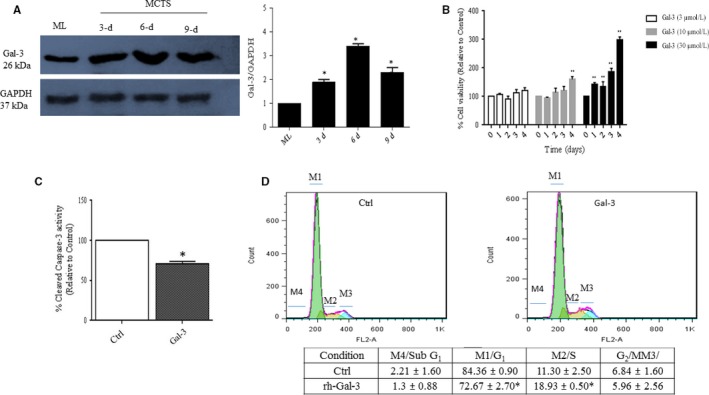
SKOV‐3 MCTS express higher Galectin‐3 expression level compared to monolayer and increase cell proliferation and survival. A, Increased Gal‐3 expression levels in SKOV3 MCTS during time compared to the monolayer. B, SKOV‐3 MCTS were treated with 3, 10, or 30 µM of exogenous Gal‐3 (rhGal‐3) for 4 consecutive days and cell viability was assessed by MTT assay. C, SKOV‐3 MCTS were incubated for 48 h with rhGal‐3 (30 µM) and Caspase‐3 activity was measured using colorimetric protease assay based on the spectrophotometric detection of the chromophore, p‐nitroaniline (p‐Na), after its cleavage from the labeled caspase substrates. D, SKOV‐3 MCTS were incubated for 48 h with rhGal‐3 (30 µM) followed by flow‐cytometry cell cycle analysis using propidium iodide (PI) staining. The percentage of cells in subG1, G0/G, S, and G2/M phases of the cell cycle was determined and showed here. Data are presented as Mean ± SD, n = 3. *: *P* ≤ 0.05; **: *P* ≤ 0.01; ***: *P* ≤ 0.001 compared to control. Gal‐3: Galectin‐3; Ctrl: control; ML: monolayer; MCTS: multicellular tumor spheroids

Increased chemoresistance has been demonstrated in MCTS^4^ and Gal‐3 was reported to be associated with higher chemoresistance.^24^ This raised the question of whether Gal‐3 expression levels differs in 3D compared to 2D cell culture model. Here, we found 2.0‐fold (*P* ≤ 0.01), 3.4‐fold and 2.3‐fold (*P* ≤ 0.001) higher Gal‐3 expression levels in 3, 6, and 9 days SKOV‐3 MCTS, respectively, compared to monolayer SKOV‐3 cell culture (Figure 1A, left and right panels). Next, we determined whether higher Gal‐3 expression levels could be associated with cell survival and proliferation. To this order, SKOV‐3 MCTS were treated with rhGal‐3 (3‐10‐30 µM) for 1, 2, 3, and 4 consecutive days, which revealed 50% increased cell viability in 4 days rhGal‐3 treated cells (*P* ≤ 0.01) and time‐dependent increased cell viability with 30 µM up to maximum 300% (*P* ≤ 0.001) in 4 days treated cells compared to untreated cells (control) (Figure 1B). Additionally, MCTS treated with rhGal‐3 (30 µM) showed decreased apoptosis as revealed by 30% (*P* ≤ 0.05) reduced level of cleaved caspase‐3 activity and increased cell proportion in S phase (18.9% vs 11.3%, *P* ≤ 0.05) (Figure 1D). Moreover, decreased cell death and increased expression levels of BCL‐2 and cyclinD1 were observed upon rhGal‐3 treatment (Figure S2A,B).

### 3.2 Gal‐3 triggers STAT3 phosphorylation

**Figure 2:**
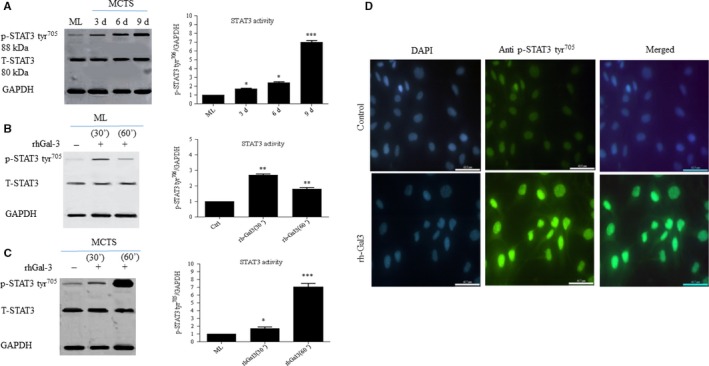
Galectin‐3 triggers STAT3 activation in 2D and 3D SKOV‐3 cell culture. A, Higher STAT3 activity as revealed by increased p‐STAT3 tyr^705 ^levels in SKOV3 MCTS during time compared to ML (left and right panels). B, Gal‐3‐induced STAT3 activity in 2D or 3D SKOV‐3 cells. Cells were treated with rhGal‐3 (30 µM) for 30 and 60 min. C, Western blot analysis of p‐STAT3tyr^705^ revealed higher Gal‐3‐induced STAT3 activity in MCTS compared to ML as shown in B (left and right panels). D, Immunofluorescence of p‐STAT3 tyr^705^ in rhGal‐3‐treated SKOV‐3 cells for 300 min. Western blots represent one of the three independent experiments. Data are presented as Mean ± SD, n = 3. *: *P* ≤ 0.05; **: *P* ≤ 0.01; ***: *P* ≤ 0.001 compared to control. Scale bar = 62.5 µm

Several studies have shown that STAT3 is constitutively activated in OC cell lines and primary human tumors and contributes to cell survival, proliferation, chemoresistance, migration, and invasion of OC cells.^11^, ^25^, ^26^ Hence, it was tempting to speculate that STAT3 activity could be differently modulated in 2D and 3D SKOV‐3 cells. Our results revealed an increased level of p‐STAT3 tyr^705 ^by 1.7‐fold (*P* ≤ 0.05), 2.4‐fold (*P* ≤ 0.05), and 7.0‐fold (*P* ≤ 0.001) in 3, 6, and 9 days SKOV‐3 MCTS, respectively, compared to the monolayer (Figure 2A, left and right panels). Next, we investigated a possible relation between STAT3 activation and Gal‐3, by treating 2D and 3D SKOV‐3 cells with exogenous Gal‐3 for 30 and 60 minutes. Western blot analysis revealed a 2.0‐fold (*P* ≤ 0.01) and 7.0 fold (*P* ≤ 0.001) increased levels of p‐STA3 tyr^705^ in the 2D and 3D SKOV3 cell culture, respectively, after 1 hour treatment with rhGal‐3 (Figure 2B,C, left and right panels). Correspondingly, immunostaining revealed increased nuclear localization of p‐STAT3 tyr^705^ in the presence of rhGal‐3 (Figure 2D).

### 3.3 Synergistic interaction of Pect‐MCP and paclitaxel to kill SKOV‐3 MCTS

**Figure 3:**
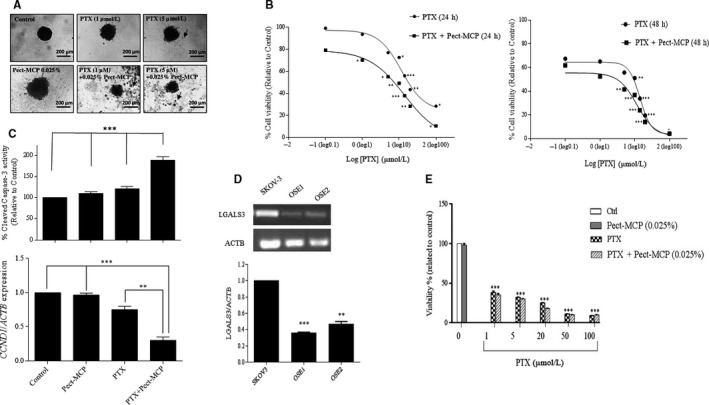
Pect‐MCP synergizes with paclitaxel to kill SKOV3 MCTS but not normal primary human ovarian surface epithelial cells. A, Photograph of SKOV‐3 MCTS treated with PTX or Pect‐MCP alone or in combination for 48 h compared to control (untreated). Floating cells represent dead cells (arrows). Photos are representative of one of the three performed experiments. B, Cells were treated, with different concentrations of PTX ranging from 0.1 µM to 100 µM alone or in combination with Pect‐MCP (0.025%) for 24 h and 48 h, respectively. Cytotoxicity was assessed by MTT assay. The synergistic effect of PTX and Pect‐MCP combination was analyzed by nonlinear regression using GraphPad Prism software (n = 4). Data are presented as mean ± SD, n = 3. C, SKOV‐3 MCTS were treated with PTX (5 µM) or Pect‐MCP (0.025%) alone or in combination and Caspase‐3 activity was measured using colorimetric protease assay as indicated (upper panel). Scale bar: 100 µm. CCND1 expression levels by SKOV‐3 MCTS were assessed by qRT‐PCR in the absence or presence of drugs alone or in combination (5 µM PTX, Pect‐MCP 0.025%) GAPDH expression levels were used as internal control and the results were analyzed by REST software (Mean ± SD, n = 3). D, Normal primary ovarian surface epithelial (OSE) cells were isolated and cultured (n = 2) as described in material and methods and Gal‐3 expression levels were significantly lower in OSE cells compared to SKOV‐3 cells as assessed by qRT‐PCR. E, OSE cells were treated with PTX with various concentrations as indicated or with Pect‐MCP (0.025%) alone or in combination for 48h and cytotoxicity was assessed by MTT assay from two independent experiments. *: *P* ≤ 0.01; **: *P* ≤ 0.01; ***: *P* ≤ 0.001 compared to control. Scale bar = 100 µm

Treatment of SKOV‐3 MCTS with PTX alone showed increased cell death at 5 µM concentration (Figure 3A, upper panel, arrow). Pect‐MCP alone has no cytotoxic effect (Figure 3A, lower panel); however, there was increased cell death in MCTS treated with PTX (1 or 5 µM) in combination with Pect‐MCP (Figure 3A, lower panel, arrows). Correspondingly, two‐way ANOVA analysis revealed a significant interaction between Pect‐MCP and applied concentrations of PTX with [F (7, 80 = 7.48, *P* < 0.0001] and [F (7, 57) = 12.91, *P* < 0.0001] after 24 and 48 hours, respectively. Further analysis by Bonferroni's posttest revealed a significant decrease in cell viability at different concentrations of PTX alone or in combination with Pect‐MCP after 24 and 48 hours (Figure 3B, right and left panels). Furthermore, the dose‐response analysis revealed almost fourfold decreased IC_50_ values with PTX + Pect‐MCP compared to PTX alone (Table 1). Correspondingly, there was almost 70% (*P* ≤ 0.001) increased caspase‐3 activity in Pect‐MCP + PTX compared to control or drugs alone (Figure 3C, upper panel) and CCND (cyclin D1) expression level was decreased by 25% and 75% (*P* ≤ 0.001) in the presence of PTX, and Pect‐MCP + PTX, respectively (Figure 3C, lower panel). It was important to show that Pect‐MCP specifically synergize with PTX to kill cancerous cells. To this order, normal primary ovarian surface epithelial cells (OSE) were isolated which showed epithelial morphology and expressed E‐cadherin, cytokeratine‐ 7 and ‐18 as well as vimentin and EpCAM (Figure S1C,D). The low expression level of LGALS3 (Galectin‐3) in OSE compared to SKOV‐3 cell line is noteworthy (Figure 3D, upper and lower panels) which may explain the lack of Pect‐MCP synergistic effect with PTX on OSE cells (Figure 3E).

**Table 1:** IC_50_ values with PTX *versus* Pect‐MCP + PTX: Effect on SKOV‐3 MCTS

Treatment	IC_50_ after 24 h Mean (95% confidence interval)	IC_50_ after 48 h Mean (95% confidence interval)
PTX	21.73 (17.78‐26.55)	4.09 (2.25‐7.43)
PTX + Pect‐MCP	5.31 (4.46‐6.32)	1.39 (0.98‐1.97)

### 3.4 Pect‐MCP and PTX in combination strongly inhibits STAT3 activity and its downstream target HIF‐1α

**Figure 4:**
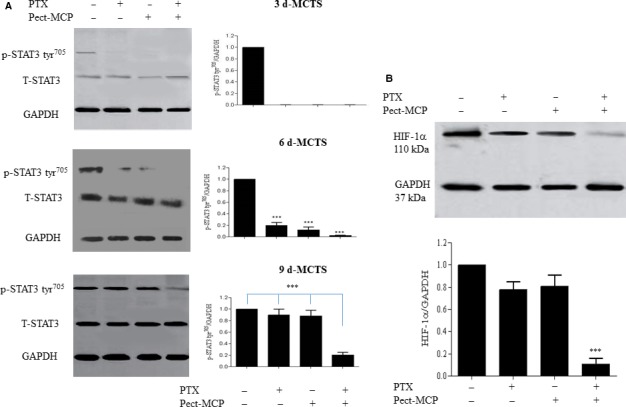
PTX and Pect‐MCP in combination strongly decrease STAT3 activity and HIF‐1α expression levels in SKOV‐3 MCTS. A, p‐STAT3 tyr^705^ levels were increased in MCTS in a time‐dependent manner which was abrogated or decreased in 6 and 9 days MCTS, respectively. B, HIF‐1α expression analysis in 9 days MCTS treated with each drug alone or in combination. Western blots represent one of the three independent experiments and results were normalized compared to GAPDH as an internal control. Mean ± SD, n = 3. *: *P* ≤ 0.01; **: *P* ≤ 0.01; ***: *P* ≤ 0.001 compared to control

Here we showed Gal‐3‐induced STAT3 activity, next we sought to determine the effect of Pect‐MCP alone or in combination with PTX on STAT3 activation in MCTS. STAT‐3 activity was detectable in control but not clearly apparent in treated cells in 3 days culture SKOV‐3 MCTS (Figure 4A, upper panel, left and right). However, in 6 days MCTS, we found 80% and 78% (*P* ≤ 0.001). Decreased p‐STAT‐3 tyr^705^ levels with PTX or Pect‐MCP, respectively (Figure 4B, middle panel, left and right), and simultaneous treatment with PTX + Pect‐MCP abrogated STAT‐3 activity (Figure 4A, middle panel, left and right) compared to control. A prominent STAT‐3 activity in 9 days MCTS was evident which were similar in control and PTX or Pect‐MCP treated cells, which was reduced by 80% in the presence of Pect‐MCP and PTX in combination (*P* ≤ 0.001) compared to other conditions (Figure 4A, lower panel, left and right). The HIF1A gene was found to contain STAT3 binding sites and STAT3 was confirmed to directly bind the HIF1A gene promoter.^27^ In order to validate the effect of PTX + Pect‐MCP on the STAT‐3 activity, we further assess the effect of these drugs on HIF‐1α as a downstream target of STAT3. Our finding showed that in 9 days MCTS culture, although not significant PTX or Pect‐MCP alone led to 15% decreased level of HIF‐1α while, combined Pect‐MCP with PTX showed 88% decreased levels of HIF1‐α (*P* ≤ 0.001) (Figure 4B, upper and lower panels).

### 3.5 Pect‐MCP antagonizes substrate‐dependent cell adhesion and reduces migration and invasion of 2D SKOV‐3 cells

**Figure 5:**
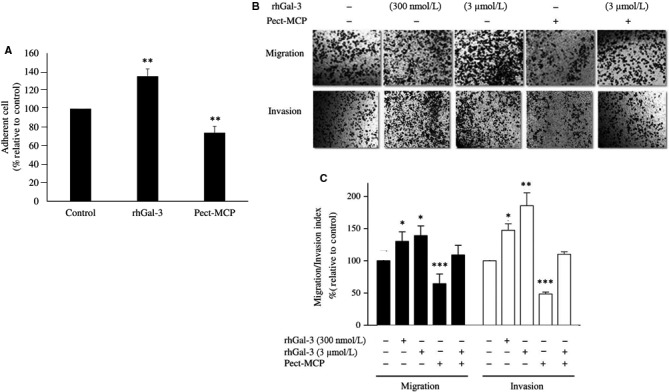
Exogenous Galectin‐3 increase cell‐substrate adhesion, migration, and invasion of SKOV‐3 cells in 2D culture. Cells were treated with rhGal‐3 (3 µM) or Pect‐MCP 0.025% alone or in combination for overnight then trypsinized and seeded at 25 × 10^3^ cells/well for further experiments. A, Cells were seeded into Matrigel (40 µg/mL) coated 96‐well plate for 15 min at 37°C. Results were expressed as a percentage of adherent cells relative to control. B, Cells were treated with rhGal‐3 with indicated concentration or with Pect‐MCP (0.025%) alone or in combination for 14 h and migration and invasion were assessed using transwells. Photos are representative of one of the three performed experiments. C, Quantification of cell migration and invasion by counting cells at 10 random fields (n = 3, Mean ± SD). Scale bar = 100 µm. *: *P* ≤ 0.05; **: *P* ≤ 0.01; ***: *P* ≤ 0.001

Extracellular Gal‐3 has been shown to enhance tumor cell adhesion to the extracellular matrix and promote cancer dissemination.^28^ Here, we investigated the effect of exogenous Gal‐3 or Pect‐MCP alone or in combination on cell adhesion to Matrigel‐coated wells. There was 30% increased SKOV‐3 cell adhesion to Matrigel‐coated wells in the presence of rhGal‐3 (*P* < 0.01); whereas, Pect‐MCP‐treated cells showed 35% decreased (*P* < 0.01) cell adhesion compared to control (Figure 5A,B). Moreover, cell migration was increased by almost 30% in the presence of rhGal‐3 (*P* ≤ 0.05) while treated cells with Pect‐MCP showed 50% decreased cell migration (*P* < 0.001) (Figure 5B,C). The effect of exogenous Gal‐3 on cell invasion was dose‐dependent which was increased by 35% (*P* ≤ 0.05) with the low concentration and 75% (*P* < 0.01) with the higher concentration of Gal‐3 compared to control (Figure 5B,C). In the presence of Pect‐MCP, there was 55% decreased cell invasion (*P* < 0.001) (Figure 5B,C). Cell migration and invasion remain unchanged upon simultaneous addition of exogenous Gal‐3 and Pect‐MCP compared to control, which showed the specific binding of Pect‐MCP to Gal‐3 (Figure 5B,C).

### 3.6 Potent inhibition of migration and invasion of SKOV3 MCTS with Pect‐MCP and PTX in combination

**Figure 6:**
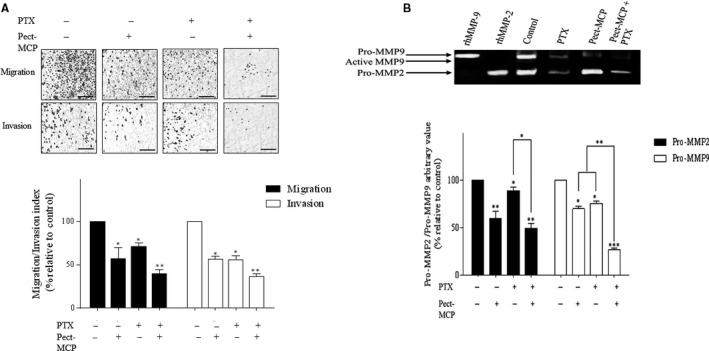
Pect‐MCP and PTX in combination inhibit SKOV‐3 MCTS migration, invasion, and reduce MMP‐2, ‐9 expression levels. A, Transwell migration and invasion assay were assessed after 14 h. Photos are representative of one of the three performed experiments. B, Quantification of cell migration and invasion by counting cells at ten random fields. C, Gelatin zymography of MMP‐2 and ‐9 of harvested conditioned media from transwell invasion assay. n = 3, Mean ± SD, *: *P* ≤  0.05; **: *P* ≤ 0.01. Scale bar = 100 µm

Epithelial ovarian cancer (EOC) does not disseminate and metastasizes through vasculature but metastasis occurs through the attachment of MCTS ovarian cancer cells to sub‐mesothelium extracellular matrix and subsequently, they invade the peritoneum and initiate metastatic tumor growth.^1^, ^2^ Here, we investigated the migration and invasion of MCTS and their subsequent MMP‐2 and ‐9 releases in the medium. MCTS migration was decreased by 45% with PTX (*P* ≤ 0.05), 30% with Pect‐MCP (*P* ≤ 0.05), and 60% with PTX + Pect‐MCP (*P* ≤ 0.01) compared to control (Figure 6A, upper and lower panels). Similarly, there was 40% decreased invasion in MCTS treated with PTX or Pect‐MCP alone (*P* ≤ 0.05) and 70% decreased invasion with PTX + Pect‐MCP (*P* ≤ 0.01) compared to control (Figure 6A, upper and lower panels).

Gelatin zymography revealed 30% and 35% decreased levels of MMP‐9 with PTX or Pect‐MCP (*P* ≤ 0.05) compared to control, respectively. There were 50% decreased levels of MMP‐9 with PTX + Pect‐MCP (*P* ≤ 0.001) compared to control (Figure 6B, upper and lower panels). Similarly, MMP‐2 levels were decreased by 40% with PTX (*P* < 0.05), 20% with Pect‐MCP (*P* ≤ 0.05) and 60% with PTX + Pect‐MCP (*P* ≤ 0.01) compared to control (Figure 6B, upper and lower panels).

### 3.7 Pect‐MCP alters integrin mRNA levels and PKB/AKT activation

**Figure 7:**
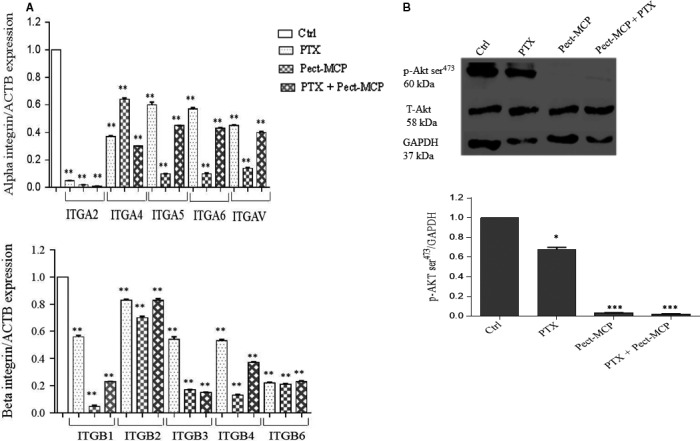
Pect‐MCP alters integrin expression and subsequent PKB/AKT activation. SKOV‐3 MCTS were treated with Pect‐MCP (0.025%) or PTX (5 µM) alone or in combination for 24 and expression levels of integrin and PKB/AKT activation was analyzed using qRT‐PCR and western blot analysis, respectively. A, Alpha (upper panel) and beta (lower panel) integrin subunit levels were assessed by qRT‐PCR. B, Western blot of p‐AKT ser^473^ represents one of three independent experiments (upper panel). Normalized values from three independent western blots (lower panel). GAPDH levels were used as an internal control. *: *P* ≤ 0.05; ***: *P* ≤ 0.001 as compared to control (Ctrl). Mean ± SD, n = 3

Integrins are involved in the various biological processes which are required for cancer initiation and progression such as cell proliferation and survival, cell adhesion, migration, and invasion.^29^ Here, we observed a significant inhibitory effect of Pect‐MCP on cell adhesion, migration, and invasion of 2D or 3D SKOV‐3 cells. These results prompted us to further investigate the effect of Pect‐MCP, PTX alone or in combination on ITGA2, ITGA4, ITGA5, ITGA6, ITGAv, ITGB1, ITGB2, ITGB3, ITGB4, and ITGB6 expression levels in SKOV‐3 MCTS. We found decreased levels of ITGA2 by more than 95% in the presence of each drug alone or in combination compared to control (*P* ≤ 0.001) (Figure 7A, upper panel).All other integrin mRNA levels were significantly decreased in the presence of each drug alone or in combination compared to control (Figure 7A, upper and lower panels). However, the inhibitory effect of Pect‐MCP on ITGA5, ITGA6, ITGAv, ITGB1, ITGB3, and ITGB4 expression levels was more potent compared to PTX alone or control (*P* ≤ 0.001)(Figure 7A, upper and lower panels). For most of the assessed integrins, lower mRNA levels were observed in the presence of Pect‐MCP + PTX compared to PTX alone (ranging from 53% to 85%, *P *≤ 0.001) (Figure 7A, upper and lower panels). However, the decreased levels of integrin expression were mainly due to the presence of Pect‐MCP as no additive effect was observed in the presence of both drugs together (Figure 7A, upper and lower panels).

It is well known that activation of the FAK‐PI3K‐AKT pathway is involved in integrin‐mediated cell adherence to the ECM, preventing cells from death.^30^ In the next step, activation of PKB/AKT was assessed in the presence of PTX, Pect‐MCP alone or in combination. We found decreased p‐AKT ser^473^ levels by 38% with PTX compared to control (*P* ≤ 0.05) (Figure 7B, upper and lower panels) which showed more than 90% decreased levels either with Pect‐MCP alone or in combination with PTX compared to control (Figure 7B, upper and lower panels). Altogether, we showed that Pect‐MCP potently decreases cell survival and thereby decreases PKB/AKT activity through alteration in integrin expression levels.

### 3.8 Gal‐3 expression level is increased in HGSOC specimens compared to normal ovary

**Figure 8:**
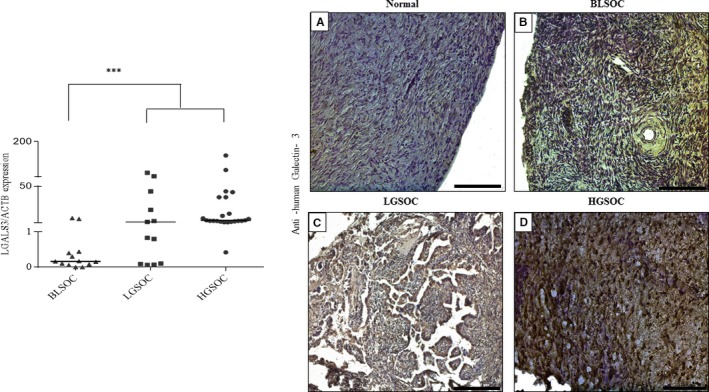
Increased expression of Galectin‐3 in human serous epithelial ovarian cancer subtypes related to normal ovary. A, Dot plot comparing the distribution of normalized qRT‐PCR analysis for LGALS3 expression levels in tumor specimens related to normal healthy ovaries. Values were normalized relative to ACTB expression levels used as an internal control. The median bar was significantly higher in LGSOC and HGSOC groups compared to the BLSOC group. B, Immunohistochemistry analysis of serous ovarian cancer specimens (n = 46) compared to the normal healthy ovary (n = 10). BLSOC (n = 12): borderline serous ovarian cancer; LGSOC (n = 12): low‐grade serous ovarian cancer; HGSOC (n = 22): high‐grade serous ovarian cancer. ***: *P* ≤ 0.001, Scale bar = 200 µm

Gal‐3 mRNA levels were higher in tumor specimens compared to the normal ovary (*P* ≤ 0.001) (Figure 8A). No Gal‐3 immunostaining was observed in normal healthy ovaries and Gal‐3 immunostaining was observed in tumor specimens (Figure 8B). However, the immunostaining intensity was variable in different tumor subtypes (Table 2). In BLSOC, 50% of samples were negative and the other 50% showed moderate Gal‐3 immunostaining (Table 2). HGSOC samples showed a higher percentage of strong immunostaining compared to LGSOC (50% vs 25%, Table 2).

**Table 2:** Immunostaining intensity of Galectin‐3 in serous ovarian cancer specimens *versus* normal ovaries

	Normal (n = 10)			BLSOC (n = 12)			LGSOC (n = 12)			HGSOC (n = 14)		
Immunostaining	0	+1	+2	0	+1	+2	0	+1	+2	0	+1	+2
Galectin‐3	10 (100)	–	–	6 (50)	6 (50)	–	1 (8.3)	8 (66.7)	3 (25)	2 (9)	9 (41)	11 (50)

Borderline serous ovarian cancer (BLSOC), Low‐grade serous ovarian cancer (LGSOC), High grade serous ovarian cancer (HGSOC) Number in parentheses represents percentage.

### 3.9 LGALS3 correlates positively with various integrin mRNA levels in different subtypes of serous EOC tumors

Since we found that Pect‐MCP could modulate integrin expression levels, next we investigate a possible relationship between LGALS3 and integrin mRNA levels in different subtypes of human serous ovarian cancer. Significant higher expression levels of ITGA2, ITGA4, ITGA6, and ITGAv were detected in HGSOC compared to normal healthy ovary or LGSOC (Figure S3A,B,D). Similarly, the mRNA levels of ITGB1, ITGB3, and ITGB6 were higher in HGSOC compared to normal healthy ovaries or LGSOC (Figure S4A,C,E). In BLSOC group, the LGALS3 expression level was significantly and positively correlated with ITGA4, ITGB4, and ITGB6 (Table 3). In LGSOC, there was a positive and significant correlation between LGALS3 and ITGA5 (Table 3) and in HGSOC, LGALS3 was positively and significantly correlated with ITGA5, ITGB2, and ITGB6 (Table 3).

**Table 3:** Correlation between LGALS3 and integrins in human serous ovarian cancer specimens

Histotype	BLSOC			LGSOC		HGSOC	
Genes	ITGA4	ITGB4	ITGB6	ITGA5	ITGB2	ITGA5	ITGB6
LGALS3	*P* = 0.033 r = 0.63	*P* = 0.070 r = 0.8	*P* = 0.026 r = 0.65	*P* = 0.080 r = 0.70	*P* = 0.035 r = 0.59	*P* = 0.040 r = 0.83	*P* = 0.044 r = 0.51

## 4 DISCUSSION

Due to the chemoresistance of ovarian cancer seminal efforts have been undertaken for sensitizing ovarian cancer cells to chemotherapy. In contrast to other cancers that spread by blood circulation, OC metastasis requires the formation of MCTS in the peritoneum and their further adherence to mesothelium. Thus, 3D cell culture models better mimic a physiological microenvironment than conventional 2D cell culture.^18^ Moreover, ovarian cancer MCTS demonstrate chemotherapeutic resistance relative to cells in traditional 2D culture.^31^

Higher expression of Gal‐3 was demonstrated in EOC patients^32^, ^33^ and other studies showed that knockout of Gal‐3 expression by RNA interference or use of a dominant‐negative form of the Gal‐3 enhanced cytotoxic effect of Paclitaxel in 2D SKOV‐3 cell culture.^8^, ^33^ In addition, Gal‐3 could mediate OC cell survival and chemoresistance through TLR4 signaling activity and NF‐kB pathway.^24^ Our results here showed that Pect‐MCP synergizes with PTX to enhance the apoptosis of SKOV‐3 MCTS which corroborates with our previous study in the 2D model.^20^

To the best of our knowledge, this report describes for the first time the higher expression of Gal‐3 in MCTS compared to monolayer ovarian cancer cells. The MCTS in ascites overcome anoikis and it has been demonstrated that Gal‐3 prevents anoikis in tumor cells.^34^ It should be noted that SKOV‐3 cells were reported to be anoikis resistant,^35^ thus it may be tempting to speculate that increased expression level of Gal‐3 could be related to anoikis pathway bypass in SKOV‐3 MCTS.

Numerous studies showed that constitutive activation of STAT3 was associated with chemoresistance in human ovarian cancer cells.^14^, ^24^, ^36^, ^37^, ^38^ Moreover, a higher level STAT3 activity was reported in 3D vs 2D SKOV‐3 cell culture.^39^ This raises the question of whether Gal‐3 could mediate chemoresistance through STAT3 activation. This study for the first time showed enhanced STAT3 phosphorylation upon the addition of exogenous Gal‐3 to ovarian cancer cells. Moreover, we report here that both increased level of Gal‐3 expression and STAT3 phosphorylation were associated with MCTS size. Other study showed that SKOV‐3 cells exhibiting endogenous STAT3 phosphorylation are more eager to form MCTS from single cells which were positively correlated with chemoresistance of SKOV‐3 cells.^38^, ^39^ To get a deeper understanding of the role of Gal‐3‐induced STAT3 activation in OC chemoresistance, we further evaluated the effect of Pect‐MCP with or without PTX on p‐STAT3 levels in SKOV‐3 MCTS. Interestingly, we found that Gal‐3‐mediated STAT3 phosphorylation was abrogated or reduced in the presence of PTX + Pect‐MCP in an MCTS size‐dependent manner. Our finding was validated by the fact that treatment of MCTS with Pect‐MCP with or without PTX showed strong downregulation of HIF1α expression as a known downstream target of STAT3. Moreover, it is important to note that Gal‐3 promoter contained conserved HIF‐1a binding motifs, and gain and loss of function analyses showed that in HIF1A null cells there was loss of Gal‐3 promoter activity while in HIF1A overexpressed cells the activity of Gal‐3 promoter activity was increased.^40^ This may suggest that higher expression of Gal‐3 in MCTS could be related to PTX resistance through increased p‐STAT3 levels and HIF‐1α may further increase Gal‐3 expression levels.

It is interesting to note that an association between Gal‐3 and STAT3 activation was found in pathological‐associated inflammation in the brain.^41^ It has been demonstrated that extracellular Gal‐3 could amplify the inflammatory cascade in the brain through the rapid induction of STAT3 phosphorylation in monolayer primary glial cell culture.^41^ It should be emphasized that inflammation plays a key role in ovarian cancer initiation and progression and one could speculate that increased Gal‐3 expression in ovarian cancer MCTS would be through paracrine effects of secreted cytokines by OC cells. We have previously shown that SKOV‐3 cells secrete different cytokines which are involved in cell migration and invasion.^22^ Moreover, it has been demonstrated that Gal‐3 upregulates cytokines expression in SKOV‐3 cells; thus we cannot exclude that in our model Gal‐3‐induced STAT3 activation could occur through cytokines.^24^ It has been demonstrated that Gal‐3 has a critical role in cell adhesion by binding to the glycoprotein component of the extracellular matrix such as laminin, fibronectin, collagen I and IV. 42 Accordingly, here, we showed that SKOV3 cell adhesion to ECM component was enhanced in the presence of rhGal‐3, while inhibition of endogenous Gal‐3 by Pect‐MCP led to reduced substrate‐dependent cell adhesion.

The integrins expressed on the surface of EOC cells are essential to the attachment of the EOC cell to the sub‐mesothelial ECM (Shield et al, 2007; Burleson et al, 2004). It has been reported that disaggregation of ovarian MCTS on type I and IV collagen, laminin and fibronectin are mediated through α2, α5, α6, and β1 integrins.^2^, ^43^, ^44^ The translational blockage of α5 and α6 integrins decreased the migratory and the invasive capacity of SKOV‐3 cells and sensitized these cells to carboplatin while blocking integrin β3 generated resistance to this drug.^45^ It should be noted that extracellular Gal‐3 could bind to integrins mainly through specific N‐terminal CRDs and promotes ligand‐induced integrin activation, thereby stimulate the proliferation, migration, and invasion of tumor cells.^46^

Here, we found that Pect‐MCP strongly reduced ITGA2, ITGA5, ITGA6, ITGAv, ITGB1, ITGB2, and ITGB3 expression levels, with subsequent decrease in PKB/AKT activity. These results may suggest that one of the mechanisms involved in Pect‐MCP sensitization of SKOV3 cells to PTX could be through alteration of integrin expression. These results have raised the question of how LGALS3 could be associated with integrin expression in different subtypes of human serous ovarian cancer. First, we found significantly higher expression of LGALS3 and ITGA2, ITGA4, ITGA5, ITGA6, ITGB1, ITGB3, and ITGB6 in HGSOC compared to other subtypes (BLSOC and LGSOC) (Figures S2 and S3). It was interesting to find that LGALS3 positively correlated with ITGB2, ITGB6, and ITGA5. The correlation of LGALS3 with ITGA5 in metastatic subtypes (LGSOC and HGSOC) and not in low‐malignant potential subtype (BLSOC) is of particular interest. It is interesting to note that α5β1 integrin was shown to be required for spheroid formation by OVCAR‐5 ovarian cancer cells which have been suggested to mediate the adhesion of ovarian cancer spheroids to extracellular matrix proteins at sites of secondary tumor growth as well as in other cell lines.^44^ In addition, other studies showed that integrin α5β1 mediates invasion and metastasis of ovarian cancer cells through activation of Akt, ERK, and JNK signaling pathways and subsequent activation of MMP9 activity.^47^, ^48^ Moreover, it has been demonstrated that α5β1 integrin played an important role in the attachment of ovarian cancer cells to mesothelium and further metastasis.^49^ Based on our data, here it is worthy to find out the mechanism of interaction between Gal‐3 and α5 integrin.

Gal‐3 is related to the migration and invasion of various types of tumor cells.^28^ It has been demonstrated that expression of Gal‐3 in LNCap cells led to increased spheroid size, migration, and invasion of these cells^50^ and increased cell migration and invasion of gastric cancer cells.^33^ In ovarian cancer, Gal‐3 silencing or use of Gal‐3c as a dominant‐negative inhibitor of Gal‐3 could inhibit the invasive and migratory capabilities of ovarian cancer cell lines and primary cancer cells.^8^, ^51^ Accordingly, here we showed that Gal‐3 inhibition by Pect‐MCP led to reduced cell migration and invasion through alteration of integrin expression and activation. It has been reported that Gal‐3 silencing in cultured human tongue cancer cells led to a decrease in MMP‐9 protein levels^52^ and interestingly, we found here that MMP‐9 was reduced in SKOV3 treated with Pect‐MCP and both MMP‐2 and ‐9 were strongly decreased in the presence of Pect‐MCP + PTX. Moreover, another study showed a relation between STAT3 activation and SKOV‐3 cell migration, invasion through modulation of MMP‐2 and MMP‐9 levels.^36^

## 5 CONCLUSION

**Figure 9:**
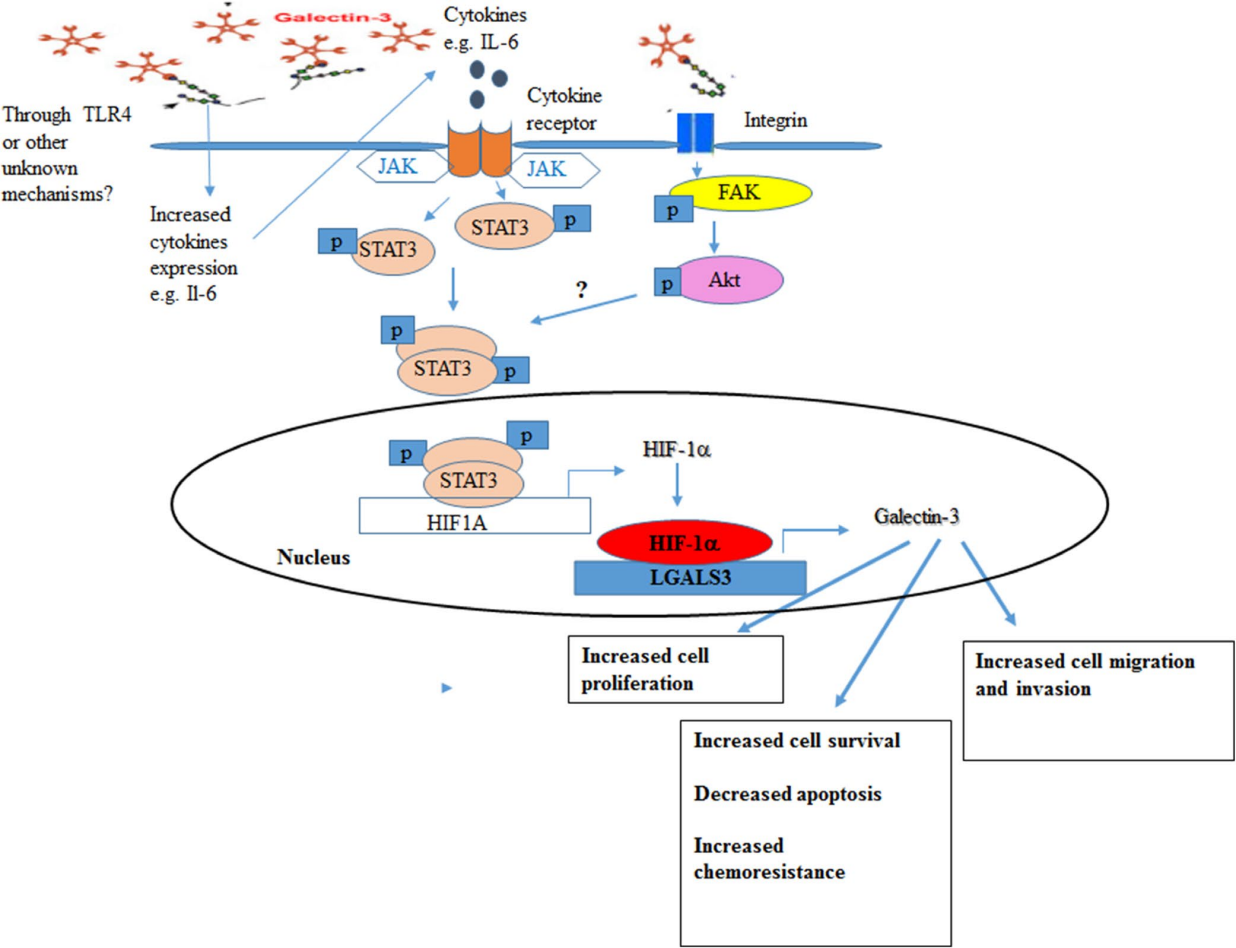
Schematic representation of Gal‐3 contribution to ovarian cancer progression through induced STAT3 activity. We hypothesize from our study here and other reports^24^, ^41^ that Gal‐3 could establish a reinforcing STAT3‐HIF‐1α positive feedback loop through cytokines (left) and/or integrins (right) leading to increased levels of Gal‐3 which results to increased cell survival, proliferation, chemoresistance, migration, and invasion of ovarian cancer cells

Taken together, this study showed for the first time that Gal‐3 expression is higher in MCTS vs monolayer ovarian cancer cells which may contribute to paclitaxel resistance through STAT3 activation. Finally, other reports showed that Gal‐3 boosts cytokine expression (eg, IL‐6, IL‐8, VEGF) in SKOV‐3 cells^24^; thus it is tempting to hypothesize that Gal‐3‐induced STAT3 activation may also occur through increased cytokine expression. A schematic diagram depicted Gal‐3 contribution to ovarian cancer progression (Figure 9) which showed that Gal‐3 could establish a reinforcing STAT3‐HIF‐1α positive feedback loop through integrins and/or cytokines which end‐up with increased levels of Gal‐3 leading to increased cell survival, proliferation, chemoresistance, migration, and invasion of ovarian cancer. Further experiments clarify Gal‐3‐induced STAT3 activity.
