# Supplementary material for: Pectasol‐C Modified Citrus Pectin targets Galectin‐3‐induced STAT3 activation and synergize paclitaxel cytotoxic effect on ovarian cancer spheroids
Source: Cancer Med. 2019 Jun 13;8(9):4315–29. doi: 10.1002/cam4.2334 (PMC6675724; doi:10.1002/cam4.2334)
Supplement: Supplementary file 1 [file CAM4-8-4315-s001.pdf]

Figure legends

Figure S1. Isolated normal primary ovarian surface epithelial cells (OSE) has epithelial morphology and express epithelial markers. OSE cells were isolated as described in material and methods. **A.** Photos showed cobble-stone epithelial morphology of OSE cells (a and b) compared to spindle-like morphology of fibroblasts in c. **B.** RT-PCR showed that OSE cells express epithelial markers (CDH1, EpCAM, KRT18 and KRT7) and to a lesser extent mesenchymal marker vimentin (VIM). Beta-2-macroglobulin (B2M) was used as positive control. Scale bar=100 μm in a and c. Scale bar=200 μm in b.

Figure S2. Exogenous Gal-3 increase SKOV-3 cell proliferation and cell survival. A. SKOV-3 MCTS were treated with rhGal-3 (30 μM) for 48h followed by BCL-2 and CCDN1 expression levels assessment by qRT-PCR. Results were normalized related to ACTB as internal control. **B.** Cell death analysis was performed in the presence or absence of rhGal-3 for 48h. Mean $\pm$ SD, n=3

Figure S3. Differential expression levels of alpha integrin subunits in BLSOC, LGSOC and HGSOC human specimens. The extracted RNA from fresh serous type EOC tumors (n = 46) were divided into three groups borderline serous ovarian cancer (BLSOC, n=12); low-grade serous ovarian cancer (LGSOC, n=12) and high-grade serous ovarian cancer (HGSOC, n=22) as well as fresh normal ovarian tissue (n =10) were analyzed by qRT-PCR for the following mRNA expression levels: (A) ITGA2, (B) ITGA4, (C) ITGA6, (D) ITGA5, (E) ITGAv. Dot plot comparing the distribution of normalized qRT-PCR analysis for afore-mentioned gene expression level in tumor specimens related to normal healthy ovaries. Values were normalized relative to ACTB expression levels used as internal control. *: $P \leq .05$; ***: $P \leq .001$ compared to control.

Figure S4. Differential expression levels of beta integrin subunits in BLSOC, LGSOC and HGSOC human specimens. The extracted RNA from fresh serous type EOC tumors (n = 46) were divided into three groups borderline serous ovarian cancer (BLSOC, n=12); low-grade serous ovarian cancer (LGSOC, n=12) and high-grade serous ovarian cancer (HGSOC, n=22) as well as fresh normal ovarian tissue (n =10) were analyzed by qRT-PCR for the following mRNA expression levels: (A) ITGB1, (B) ITGB2, (C) ITGB3, (D) ITGB4, (E) ITGB6. Dot plot comparing the distribution of normalized qRT-PCR analysis for afore-mentioned gene expression level in tumor specimens related to normal healthy ovaries. Values were normalized relative to ACTB expression levels used as internal control. *: $P \leq .05$; **: $P \leq .01$; ***: $P \leq .001$ compared to control.

Table S1. Clinicopathological characteristics of patients

Tumor grade	n	Median age	Treatment
Normal ovary	10	53	-
BLSOC	12	42	None
LGSOC (Grade I)	7	55	None
LGSOC (Grade II)	5	47	None
HGSOC (Grade III)	12	56	None
HGSOC (Grade lV)	10	61	None

Borderline serous ovarian cancer (BLSOC), Low-grade serous ovarian cancer (LGSOC), High-grade serous ovarian cancer (HGSOC).

Table S2. Primer sequences used for qRT-PCR in this study

Genes	Primer sequences
ACTB	F: 5´- CTTCCTTCCTGGGCATG-3'<br>R: 5´- GTCTTTGCGGATGTCCAC-3'
B2M	F: 5´- CCTGAATTGCTATGTGTCTGGG -3 '<br>R: 5´-TGATGCTGCTTACATGTCTCGA-3'
LGALS3	F: 5´CCTCGCATGCTGATAACAATTCT-3'<br>R: 5´TGACTCTCCTGTTGTTCTCATTGAA-3'
ITGB1	F: 5′- GTGGGTGGTGCACAAATTC-3′<br>R: 5′-GGTCAATGGGATAGTCTTCAGC-3′
ITGB2	F: 5′-TTCGGGTCCTTCGTGGACA-3'<br>R: 5′- ACTGGTTGGAGTTGTTGGTCA-3'
ITGB3	F: 5′- AGCCAACAACCCACTGTA-3'<br>R: 5′- CTGACATTCTCCCAACCTAC-3'
ITGB4	F: 5′-TGGAAGTACTGTGCCTGCTG-3'<br>R: 5′-TGCATGTTGTTGGTGACCTT-3'
ITGB6	F: 5′- TCCATCTGGAGTTGGCGAAAG-3'<br>R: 5′- TCTGTCTGCCTACACTGAGAG-3'
ITGA2	F: 5′- TAGCGCTCAGTCAAGGCATT-3'<br>R: 5′- GCACTGCATAGCCAAACTGT-3'
ITGA4	F: 5′- AGCCCTAATGGAGAACCTTGT-3'<br>R: 5′- CCAGTGGGGAGCTTATTTTCAT-3'
ITGA5	F: 5'- TTTATCGGTCTCGGGAGTTG-3'<br>R: 5'-CTTCAACTTAGACGCGGAGG -3'
ITGA6	F: 5'- TTTATCGGTCTCGGGAGTTG-3'<br>R: 5'- GGCCACTGAATGTTCAAGGT-3'
ITGAV	F: 5'- GCAACAGGCAATAGAGAT-3'<br>R: 5'- TGCTGAATCCTCCTTGACAA-3'
EPCAM	F: 5'- CCATGTGCTGGTGTGTGAAC -3'<br>R: 5'-CCTTCTGAAGTGCAGTCCGC -3'
CDH1	F: 5'- CAGGAGTCATCAGTGTGGT -3'<br>R: 5'- GGAGGATTATCGTTGGTGTCAG -3'
KRT7	F: 5'- TCCGCGAGGTCACCATTAAC-3'<br>R: 5'- GCTCTGTCAACTCCGTCTCAT-3'
KRT18	F: 5' - GGAGGCATCCAGAACGAGAA- 3'<br>R: 5' – CCAGCTGCAGTCGTGTGATA- 3'
VIM	F: 5' - GGCTCGTCACCTTCGTGAAT - 3'<br>R: 5' – GAGAAATCCTGCTCTCCTCGC - 3'

Figure S1

A

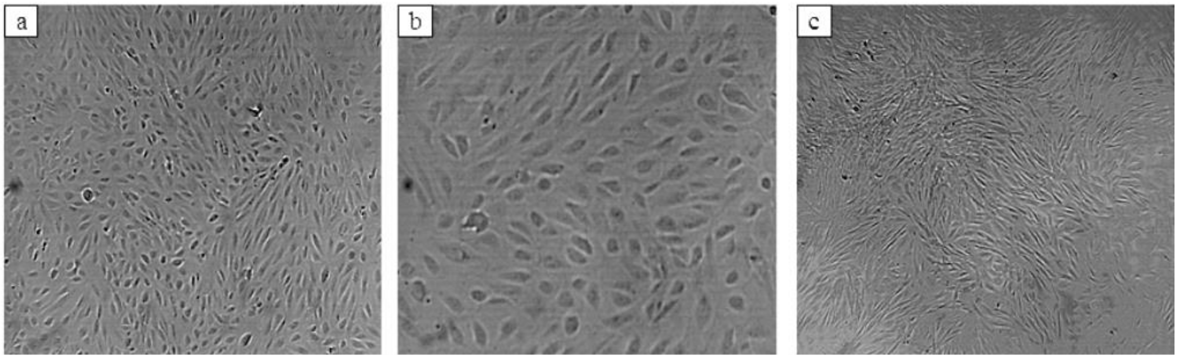

B

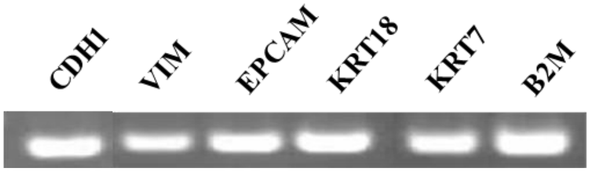

Figure S2

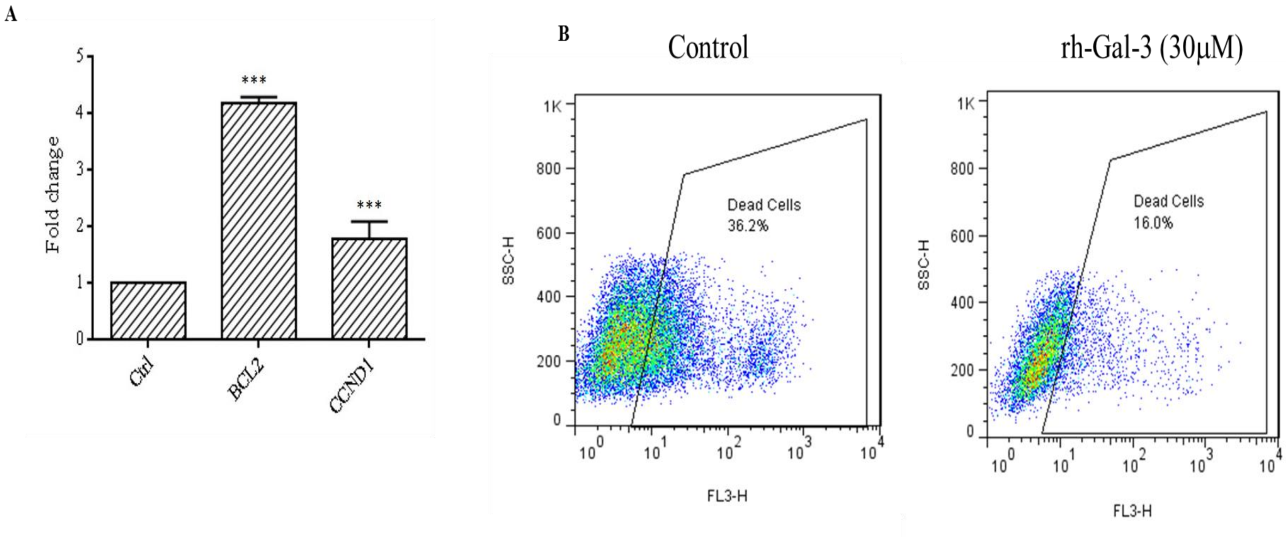
A
Fold change
***
***
Ctrl
BCL2
CCND1
B
Control
rh-Gal-3 (30µM)
Dead Cells
36.2%
Dead Cells
16.0%
SSC-H
FL3-H

Figure S3

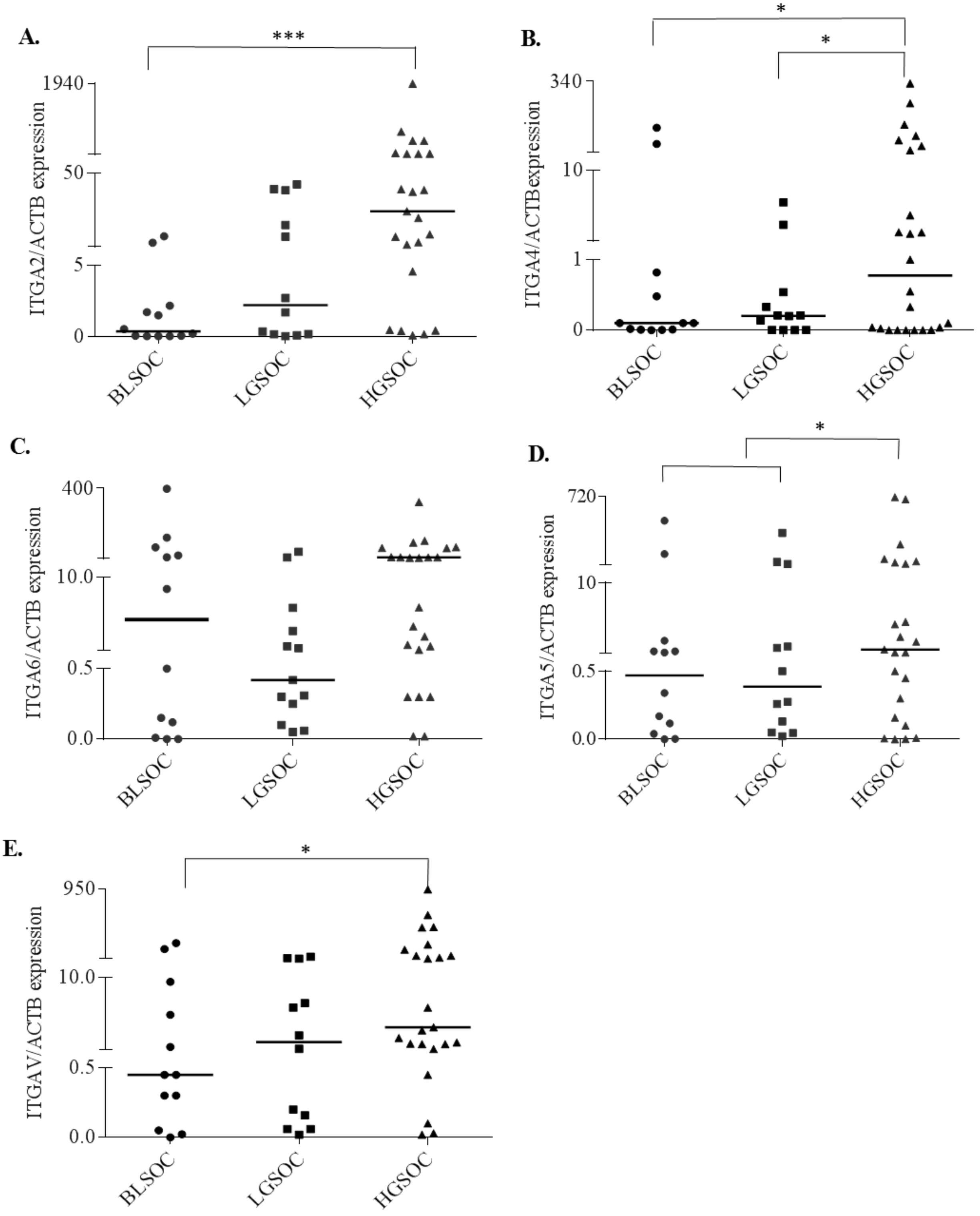
A.
***
1940
50
5
0
ITGA2/ACTB expression
BLSOC
LGSOC
HGSOC
B.
*
*
340
10
1
0
ITGA4/ACTBexpression
BLSOC
LGSOC
HGSOC
C.
400
10.0
0.5
0.0
ITGA6/ACTB expression
BLSOC
LGSOC
HGSOC
D.
*
720
10
0.5
0.0
ITGA5/ACTB expression
BLSOC
LGSOC
HGSOC
E.
*
950
10.0
0.5
0.0
ITGAV/ACTB expression
BLSOC
LGSOC
HGSOC

Figure S4

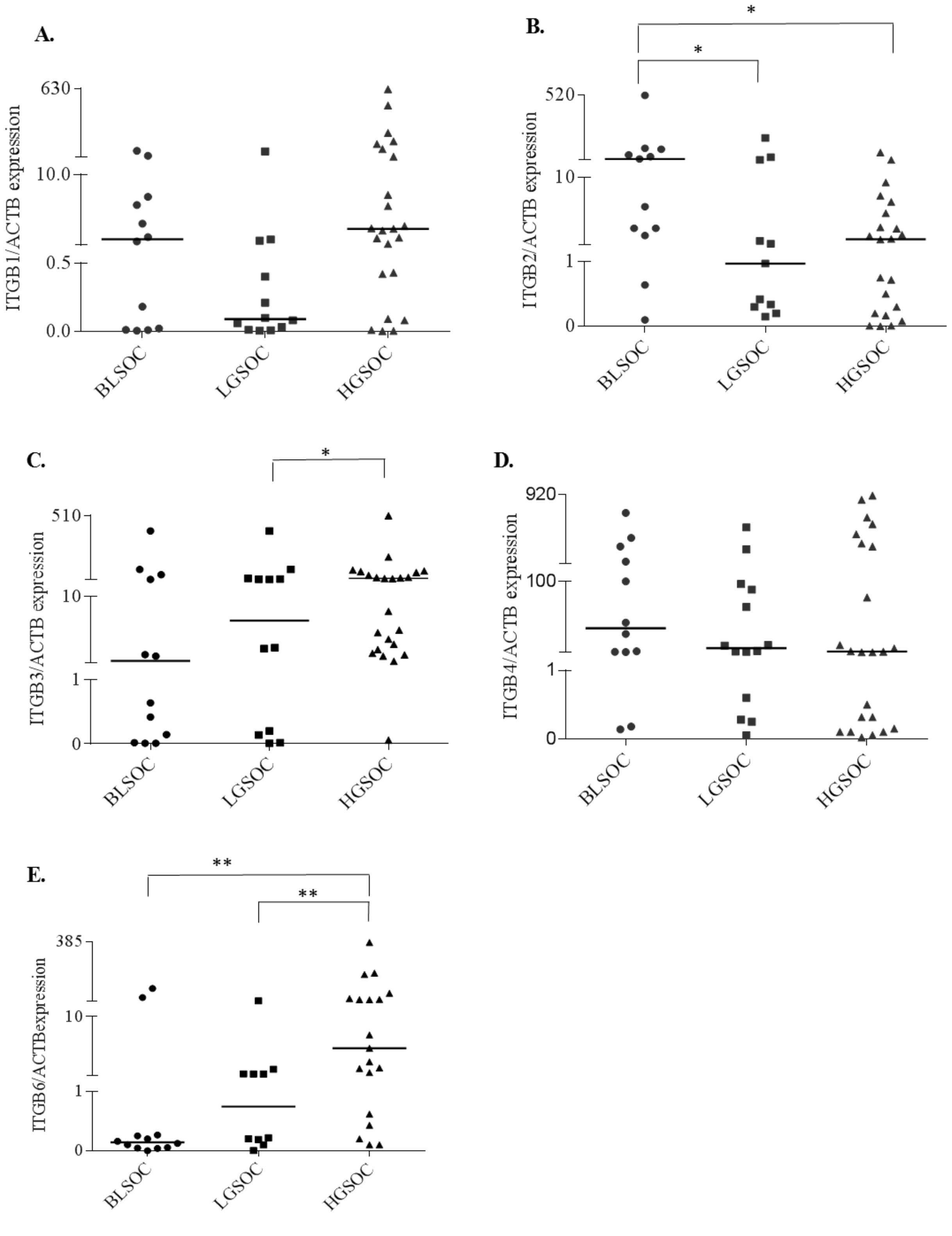
A.
ITGB1/ACTB expression
630
10.0
0.5
0.0
BLSOC
LGSOC
HGSOC
B.
*
*
ITGB2/ACTB expression
520
10
1
0
BLSOC
LGSOC
HGSOC
C.
*
ITGB3/ACTB expression
510
10
1
0
BLSOC
LGSOC
HGSOC
D.
ITGB4/ACTB expression
920
100
1
0
BLSOC
LGSOC
HGSOC
E.
**
**
ITGB6/ACTBexpression
385
10
1
0
BLSOC
LGSOC
HGSOC
